# Is Using the Strengths and Difficulties Questionnaire in a Community Sample the Optimal Way to Assess Mental Health Functioning?

**DOI:** 10.1371/journal.pone.0144039

**Published:** 2016-01-15

**Authors:** Sharmila Vaz, Reinie Cordier, Mark Boyes, Richard Parsons, Annette Joosten, Marina Ciccarelli, Marita Falkmer, Torbjorn Falkmer

**Affiliations:** 1 School of Occupational Therapy and Social Work, Curtin University, Perth, Western Australia, Australia; 2 School of Psychology and Speech Pathology Curtin University, Perth, Western Australia, Australia; 3 School of Pharmacy, Curtin University, Perth, Western Australia, Australia; 4 School of Education and Communication, CHILD programme, Institution of Disability Research Jönköping University, Jönköping, Sweden; 5 Rehabilitation Medicine, Department of Medicine and Health Sciences (IMH), Faculty of Health Sciences, Linköping University & Pain and Rehabilitation Centre, UHL, County Council, Linköping, Sweden; Philipps University Marburg, GERMANY

## Abstract

An important characteristic of a screening tool is its discriminant ability or the measure’s accuracy to distinguish between those with and without mental health problems. The current study examined the inter-rater agreement and screening concordance of the parent and teacher versions of SDQ at scale, subscale and item-levels, with the view of identifying the items that have the most informant discrepancies; and determining whether the concordance between parent and teacher reports on some items has the potential to influence decision making. Cross-sectional data from parent and teacher reports of the mental health functioning of a community sample of 299 students with and without disabilities from 75 different primary schools in Perth, Western Australia were analysed. The study found that: a) Intraclass correlations between parent and teacher ratings of children’s mental health using the SDQ at person level was fair on individual child level; b) The SDQ only demonstrated clinical utility when there was agreement between teacher and parent reports using the possible or 90% dichotomisation system; and c) Three individual items had positive likelihood ratio scores indicating clinical utility. Of note was the finding that the negative likelihood ratio or likelihood of disregarding the absence of a condition when both parents and teachers rate the item as absent was not significant. Taken together, these findings suggest that the SDQ is not optimised for use in community samples and that further psychometric evaluation of the SDQ in this context is clearly warranted.

## Introduction

### Methodological difficulties in the assessment of mental health problems in adolescence

Mental health problems are relatively common in children and youth. More than 75% of all cases of severe mental illnesses are estimated to occur prior to the age of 25 years [1,2]. Australian estimates suggest a prevalence of 14% mental illness in the 4–17 year age bracket [3]; only one in four of the identified cases were receiving professional help [3]. Mental disorders account for around 22% of all disability-adjusted life years lost in established market economies such as Australia [4]. Early detection of mental health problems in children and youth is crucial, as evidence shows that left undetected, mental health problems tend to increase in severity with age and could be antecedents of chronic, complex, disabling and expensive complications in adult life [1,5–7]. Current screening methods rely on children and youth displaying certain symptoms, or impairments in everyday functioning, in order to identify them to be at risk and in need of further evaluation and potential treatment [8].

Children commonly rely on adults in their close environment to recognise their mental health problems [9]; the most common adults being their parent or teacher. Parent-reported barriers to accessing children’s mental health care can be categorised into: (i) structural barriers (e.g., lack of providers, long waiting lists, insurance or monetary constraints, transportation problems); (ii) identification barriers (i.e., parents’, teachers’, and medical care providers’ inability to identify children’s need for mental health services; denial of the severity or need for treatment of problem); and (iii) barriers related to perceptions about mental health services (i.e., stigma related to seeking help, lack of trust in or negative experience of service providers) [9,10]. Furthermore, parents who reported barriers were more likely to have parent stressors, schedule constraints, and to be divorced compared with parents who did not report barriers[10]. Given these barriers, parents frequently seek teachers’ opinions of their child’s mental health functioning prior to contacting formal health care services [11]. Consequently, teachers have been recognised as an increasingly important stakeholder in detecting mental health problems in children and supporting child mental health [12,13].

Available research examining teachers’ abilities to detect mental health problems in their students suggest that teachers tend to have low confidence in their ability to recognise and support students’ mental health problems and their knowledge base of mental health [12–14]. Moreover, teachers tend to seek help for students with behaviours that are disruptive to other students and as a result affect their academic performance, rather than students with internalising problems [11,14]. Studies that compare parents’ abilities with teachers’ abilities to detect mental health problems in children suggest that parents usually rate their child’s problems as more important and severe than teachers [15]. As a result, mental health professionals tend to regard teachers’ reports on hyperactivity, for example [16], and mothers’ report on conduct and internalising problems to be most reliable.

To date, in the absence of gold standard measures for assessing mental health problems in children and youth, a multi-informant multimodal approach is couched as best-practice [17–22]. The literature has consistently demonstrated that informants are inconsistent in their assessment of child and adolescent mental health functioning, irrespective of the method of clinical assessment [17,18,23–26]. For example, a recent review on the psychometric properties of one of the most widely used mental health screening tool in children and youth, namely the Strength and Difficulties Questionnaire [27], reported poor to moderate weighted mean parent-teacher (inter-rater) agreement correlations (total difficulties = 0.44; hyperactivity/inattention = 0.47; emotional symptoms = 0.28; conduct problems = 0.34; peer problems = 0.35) [28]. Even when attempts were made to reduce informant discrepancies through mitigation by a senior clinician; ratings have, at best, resulted in modest levels of agreement (*r* = 0.19–0.52)[29].

Disagreements between parents’ and teachers’ ratings of a child’s behaviour could be explained by the fact that children behave differently in different contexts [15,30]. Hence, these discrepancies may reflect variation in the circumstances under which the child expresses disruptive behaviour symptoms [24]. It is also likely that parents and teachers use different benchmarks when evaluating these behaviours. For example, teachers’ ratings may be influenced by the level of difficulties experienced by the child in relation to those of other children in the class, whilst comparisons with siblings might have more bearing on parents’ ratings. Also, teachers are exposed to a large number of children and hence, a much wider and diverse comparison group [15]. Teachers may therefore be better equipped than parents to distinguish behaviours that are symptomatic of mental health problems. Thus, informant discrepancies may reflect biases in reporting, measurement error, or variability in symptomatology across settings [24].

The pattern of agreement-disagreement between parents’ and teachers’ ratings of a child’s mental health can provide a more holistic description of the child as it combines different views [30]. If so, the pattern of agreement-disagreement may give an insight into how each informant provides multidimensional information that reflects the child’s functioning in different contexts. Further research into the pattern of agreement-disagreement between informant ratings at item response level could shed some light into the cause of discrepancy [30]. The current study set out to critically examine the pattern of agreement-disagreement between parents’ and teachers’ ratings of early adolescents’ mental health functioning using a community used screening tool—the Strength and Difficulties Questionnaire (SDQ) [27].

### The tool at the centre of enquiry: The Strength and Difficulties Questionnaire (SDQ)

The Strength and Difficulties Questionnaire (SDQ) is a short, user friendly, easy to use measure of competencies and problem behaviours of children and youth [27,28]. The SDQ items and subscales were developed with reference to the main nosological categories recognised by contemporary classification systems of child mental disorders such as the Diagnostic and Statistical Manual of Mental Disorders, 4^th^ edition (DSM- IV) [31] and the International Classification of Diseases, 10^th^ edition (ICD—10) [32]. The questionnaire consists of 25 screening items that measure both psychosocial problems (i.e., emotional problems, conduct problems, hyperactivity–inattention, and peer problems) and strengths (i.e., prosocial behaviour) in children and youths aged 3–16 years [27,33,34]. It has an impact supplement that assesses chronicity, distress, social impairment and burden to others. Three dimensions of impact can be calculated; namely, perceived difficulties (is there a problem), impact score (distress and social incapacity on the child), and a burden rating (do symptoms impose a burden) [33].

The SDQ uses a multi-informant approach and is suitable for use in studies involving general community populations, in which the majority of children are healthy. Having multiple informants reporting on the SDQ is valuable due to the situational nature of psychosocial problems, particularly in children [35–37]. There are informant-rated versions, which can be completed by either the parents or teachers of children and adolescents aged 2–4 years, 4–10 years and 11–17 years; and a self-report version, which can be completed by adolescents aged 11–17 years. The present study employed the parent and teacher SDQ and impact supplement for 11–17 year olds.

#### Psychometric quality and utility of screeners–understanding relevant indices

Although the psychometric quality of mental health screeners has yet to be evaluated, quality measures should demonstrate adequate reliability and validity [8,38]. A recent literature review drawing on the psychometric properties of the parent and teachers versions of the SDQ in 4 to 12-year olds reported satisfactory pooled internal consistency for the total difficulties score (parent: *α* = 0.80; N = 53,691; teacher: *α* = 0.82; N = 21, 866) [28]. All subscales of the parent version reported internal consistency values below the recommended benchmark (prosocial behaviour: *α* = 0.67; emotional problems: *α* = 0.66; conduct problems: *α* = 0.58; and peer problems: *α* = 0.53), with the exception of the hyperactivity-inattention subscale (*α* = 0.76) [28]. All subscales of the teacher version were reported to have adequate internal consistency, with the exception of the peer problems subscale (*α* = 0.63). This means that despite the SDQ being used widely in practice and research, caution ought to be exercised when using SDQ subscales that do not meet the recommended reliability guidelines. The inter-rater agreement between parent and teacher ratings of the SDQ from eight studies by weighted mean correlations range from 0.26–0.47 [28].

#### Use of SDQ as a mental health screener

Another important characteristic of a screening tool is its discriminant ability; that is, the measure’s accuracy to distinguish between those with and without mental health problems. The SDQ has been used in epidemiological, developmental, and clinical research in many countries and has been translated into more than 60 languages [28,39–44]. The increased use of the SDQ has been accompanied by increased research on its psychometric properties. Validation studies on the SDQ have used community-based [42,43,45–48] and clinical samples [37,49,50].

The evidence to date on the discriminative ability (i.e., screening ability) of the parent and teacher versions of the SDQ in detecting mental health problems, is better in clinical samples when compared to community populations [28]. For example, combined parent and teacher reports in UK samples have been shown to have sensitivity values of 62.1% and 82.2%, in detecting mental health disorders in community and clinical samples, respectively [36,39]. When only parent reports were used, sensitivity dropped to 29.8% in a community sample and to 51.4% in the clinical sample [36,39]. In the case of only using teacher reports, sensitivity values of 34.5% and 59.8% have been documented in community and clinical samples, respectively [36,39]. The SDQ sensitivity was lowest for detecting anxiety in community samples (parent and teacher combined = 45.4%; parent = 38.8%; teacher = 15.9%). Positive predictive value (PPV) in a community sample has been shown to range from 35% (hyperactivity disorders) to 86% (emotional disorders) and negative predictive value (NPV) ranged from 83% to 98% [36].

The level of agreement between SDQ generated diagnoses (multi-informant format, parent, teacher and self-report) and clinical team diagnoses made by a community child and adolescent mental health service (regarded as gold standard) in an Australian sample has been found to be moderate; ranging from 0.39 to 0.56 [49]. The level of agreement (Kendall’s Tau-b) between the SDQ generated diagnoses and the independent clinician’s diagnoses were low to moderate in range (emotional problems, *r* = 0.26, to 0.43, hyperactivity disorder). Concurrent agreements between clinical team and the independent clinician ratings were higher (emotional problems, *r* = 0.45, to 0.65, hyperactivity disorder). The probable or 90% dichotomisation system was used to measure sensitivity of SDQ diagnoses. The ‘*probable’* dichotomisation level classifies approximately 90% of a population-based sample as having a negative test, while the ‘possible’ dichotomisation level gives a ‘test negative’ for approximately 80% of the same sample [51] The sensitivity of SDQ diagnoses was generated using the probable or 90% dichotomisation was 36% for emotional disorders, 44% for hyperactivity disorders, and 93% for conduct disorders. In contrast, the sensitivity of combined possible and probable SDQ diagnoses was 81% for emotional disorders, 93% for hyperactivity disorders, and 100% for conduct disorders. False negatives; that is, children who had a definite disorder but who were rated unlikely by the SDQ algorithm (multi-informant format), were rare for conduct disorders (n = 0, N = 130) and hyperactivity disorders (n = 2, N = 130), but more frequent for emotional disorders (n = 7, N = 130) [49].

The SDQ screening accuracy works best when it is completed by all three informants; namely parents, teachers and young people aged 11 years and older [36]. If it is impractical or uneconomical to obtain data from all informants, parents’ and teachers’ reports have been shown to have equal predictive value, although their relative value depends on the type of mental health problem [36]. The screening accuracy of the SDQ varies by mental health problem and rater [36]. For conduct and hyperactivity disorders, self-report data are of less screening value than data from either parents or teachers. In the case of emotional disorders, self-report information are about as useful as teacher data, but less useful than parent data [36]. Consequently, the parent and teacher report combinations are most often used in research [28].

In summary, although the SDQ is labelled as the most widely used screening measure of mental health problems in children and youth; the parent and teacher versions of the measure have poor concordance, questionable internal consistency, and inadequate sensitivity in a community sample. No study to date has examined the inter-rater agreement and screening concordance of the parent and teacher versions of the SDQ at *item-level* with the view of identifying the items that have the most informant discrepancies; and determining whether the concordance between parent and teacher reports on some items has the potential to influence decision making. In addressing the gap, this study aimed to:

1. examine the reliability of the teacher and parent versions of the SDQ in a community sample of young adolescents;
2. explore the inter-rater agreement-disagreement between parent and teacher ratings on the SDQ both at scale, subscale, and item level; and
3. identify whether concordance between parent and teacher reports on the SDQ (scales and subscales, and items) has the potential of identifying young adolescents *at-risk* of mental health problems.

## Method

### Participants

This cross-sectional study is part of a larger longitudinal study concerning the factors associated with student adjustment across the primary-secondary transition [52,53]. Parent and teacher reports of the mental health functioning of a community sample of 299 students with and without disabilities from 75 different primary schools across metropolitan Perth and other major city centres across Western Australia were collected. The study's cohort comprised 29% Catholic Education, 47% Government, and 24% Independent schools, which was different to the profile of all schools in Western Australia at the time of data collection (15%, 72%, and 13%, respectively). The school post code was used to calculate its socio-economic index (SEIFA Index), using the Commonwealth Department of Education, Employment, and Workplace Relations measure of relative socio-economic advantage and disadvantage [54]. The SEIFA decile was used as the measure of mean school socioeconomic status (SES), with a lower decile number meaning that the school was located in an area that was relatively more disadvantaged than other areas. Approximately 35% of the sample came from schools that fell into the 1–8 decile bands; 44% came from schools in the 9^th^ decile band; and 21% came from schools in band 10. This means that the sample was over-representative of higher SES band schools.

The mean age of the students was 11.9 years (*SD* = 0.45 years, median = 12 years). There was a nearly even split by gender (boys = 48.2%; *n* = 144). Household income data from 294 families were retrieved. The majority of the students (60%, *n* = 179) came from mid-range households; under one-third of the students (30.3%, *n* = 89) were from high-SES households and 8.8% (*n* = 26) were from low-SES groupings [55]. Informed written consent was obtained from school principals, parents, and teachers. All participants were made aware that they were not obliged to participate in the study and were free to withdraw from the study at any time without justification or prejudice. Ethics approval was obtained from Curtin University Health Research Ethics Committee in Western Australia (WA) (HR 194/2005).

### Measurement tool: The Strength and Difficulties Questionnaire (SDQ)

The 25-item teacher and parent versions of the Strengths and Difficulties Questionnaire (SDQ) were used to record each informant’s perception of four problem domains/subscales and one pro-social domain/subscale (each consisting of five items) [27,33,34]. The problem subscales include emotional symptoms, conduct problems, hyperactivity/inattention, and peer problems. Each item on the SDQ is scored on a 3-point ordinal scale with 0 = not true; 1 = somewhat true; and 2 = certainly true, with higher scores indicating larger problems (except in the case of pro-social behaviour in which a higher score indicates more positive behaviour). The SDQ total difficulties score is computed by summing the four problem behaviour subscales. Subscale scores range from 0–10, while the total difficulties SDQ score ranges from 0–40.

## Statistical Analyses

Data were managed and analysed using SPSS Version 20.0 and SAS Version 9.2 software packages. Less than 0.8% of data were missing at scale levels. The estimation maximisation algorithm and Little’s chi-square statistic revealed that the data were missing completely at random [56,57]. Replacement of missing data was undertaken using the guidelines recommended by the SDQ developers wherein, if at least three of the five SDQ items in a scale were completed, the remaining two scores were replaced by their mean. When more than three items were missing in a scale level, scores were excluded from the analysis. Independent samples *t-*tests confirmed that the profiles of those whose data were missing for various questions were similar to those who responded. The following analyses were undertaken:

1. Descriptive overview: Means and standard deviations were calculated to provide a descriptive overview of the problems reported by both parents and teachers.
2. The nature of agreement of teachers’ scores relative to parents (gold standard) was measured using the Bland-Altman Limits of Agreement (LOA) plots [58–60]. The LOA are based on the normal distribution, and bracket approximately 95% of differences between the ratings of teachers and parents. The plot of difference against mean was used to investigate any possible relationship between the measurement error and the true value for each total score.
3. Intraclass Correlation Coefficient [ICC, Absolute agreement]: To attain interval level scores for each participant and each SDQ item, parent and teacher SDQ raw scores were subjected to Rasch analysis using the Winsteps programme (version 3.70.0.2) [61]. The Rasch model enables the researcher to examine simultaneously: (i) whether or not the items define a single unidimensional construct (strengths and difficulties in this instance); (ii) the relative difficulty of each test item; and (iii) the relative strengths and difficulties score of each person [62]. In addition to estimates of the relative difficulty of items and abilities of people, the Rasch analysis yields goodness-of-fit statistics expressed in mean square (MnSq) and standardised values. Prior to further calculations, we examined the goodness-of-fit statistics for people and items to ensure that they were within an acceptable range set a priori (MnSq < 1.4; standardised value < 2) [62]; this ensured that the measured scores were true interval-level measures. The resulting person and item measure scores were then entered into SPSS to test if the data were normally distributed (using the Kolmogorov-Smirnov test for normality). As the data were normally distributed, individual child and item inter-rater reliability between parent and teacher ratings of the children’s mental health status (using the SDQ) was calculated using ICC (2,1) [absolute agreement, two-way random effects model, single measures]. Reliability refers to the degree to which participants can be distinguished from each other, despite measurement error [63]. An ICC between 0.4 and 0.7 is generally taken to indicate fair agreement, while values higher than this indicate excellent agreement [64].
4. Percentage of agreement, and overall classification accuracy index at item level, using the raw data
5. Cohen’s weighted Kappa coefficient, percentage of agreement, and overall classification accuracy index at scale level (using the *“possible or 80% dichotomisation system”*): The diagnostic algorithm was used to allocate the subscales and total SDQ scale scores into three categories and indicate the risk of difficulties, namely: ‘unlikely’, ‘possible’ and ‘probable’[51]. The weights for the calculation of weighted Kappa were obtained from the column scores using the Fleiss-Cohen method (SAS v 9.2). Essentially, more weight is given to measurements that are in closer agreement. To calculate the values for screening efficiency in terms of sensitivity, specificity, positive predictive value (PPV), negative predictive value (NPV), positive likelihood ratio (LR^+^), negative likelihood ratio (LR^-^), and diagnostic odds ratio (OR^D^), the three risk categories were reduced to two categories (‘test negative’, and ‘test positive’) [37,49]. In the first instance, the categories unlikely and possible were labelled ‘test negative’, and the third category probable was labelled ‘test positive’ (hereafter, referred to as the *“probable dichotomisation”)*. In the second calculation, only the category unlikely was labelled ‘test negative’, and the second and third categories, possible and probable, were labelled ‘test positive’ (hereafter referred to as the *“possible or 90% dichotomisation system”*).
6. Cohen’s weighted Kappa coefficient, percentage of agreement, and overall classification accuracy index at scale level (using the “probable or 90% dichotomisation system”).
7. Screening efficiency of teachers’ ratings relative to parents’ ratings, using the “probable or 90% dichotomisation” at item level.

## Results

### Descriptive overview of the reported symptoms in the students

The mental health scores of the students in the current study were better than Australian population norms for the 7–17 year old age group [51]. The internal consistencies of the total SDQ scale in the current sample were below recommended standards for reliable use in a clinical setting [64]. The parent version of the hyperactivity/ inattention subscale merely met the benchmark criterion (Table 1).

**Table 1:** Descriptive overview of mean scores by informant*.

SDQDomains	Students in the current study (N = 299)		Population norms (N = 910) [51]		Reliability of subscales in the current population (N = 299)		Weighted mean internal consistency results on the SDQ specified by informant [28] (Parent = 53,691; Teacher = 21,866)	
	Parent rating M (SD)	Teacher rating M (SD)	Parent rating M (SD)	Teacher rating M (SD)	Parent rating Cronbach’s α	Teacher rating Cronbach’s α	Parent rating Cronbach’s α	Teacher rating Cronbach’s α
**Conduct problems**	0.87 (1.22)	0.69 (1.42)	1.5 (1.6)	1.0 (1.5)	0.59	0.76	0.58 (0.46–0.76)	0.70 (0.63–0.84)
**Hyperactivity-inattention**	2.56 (2.25)	2.03 (2.35)	3.1 (2.4)	2.5 (2.6)	0.80	0.76	0.76 (0.58–0.85)	0.83 (0.66–0.89)
**Emotional problems**	1.85 (1.98)	1.17 (1.74)	2.1 (2.0)	1.4 (1.7)	0.73	0.71	0.66 (0.60–0.76)	0.73 (0.63–0.80)
**Peer problems**	1.42 (1.78)	1.27 (1.75)	1.6 (1.9)	1.6 (1.8)	0.68	0.65	0.53 (0.30–0.76)	0.63 (0.35–0.77)
**Total difficulties**	6.69 (5.42)	5.17 (5.46)	8.2 (6.1)	6.5 (6.0)	0.77	0.67	0.80 (0.53–0.84)	0.82 (0.62–0.85)

*Note*.

* The pro-social subscale has not been reported at subscale level

### Measure of the nature of agreement of teachers’ scores relative to parents (gold standard), using Bland—Altman Limits of Agreement (LOA) plots

**Fig 1:**
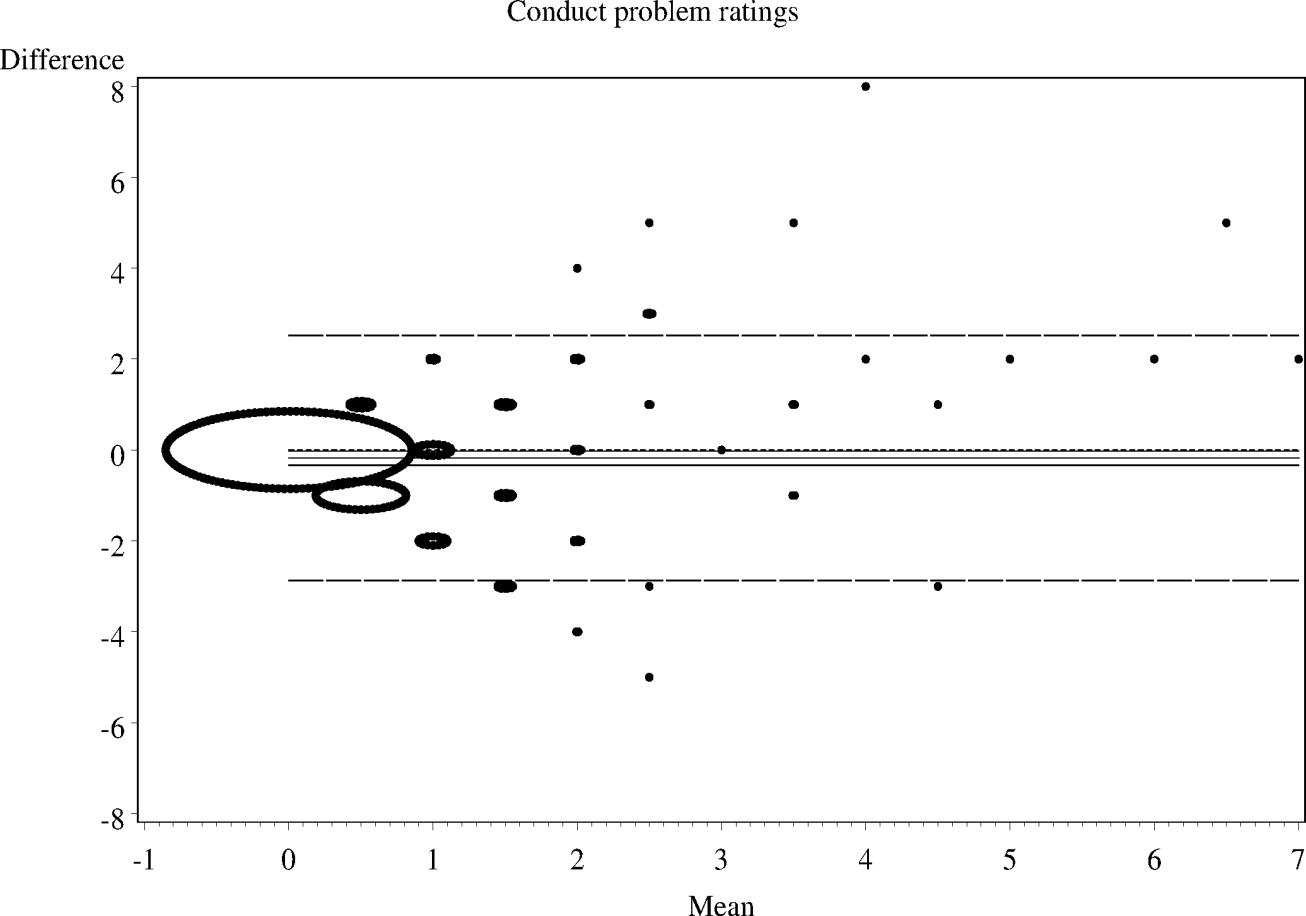
Bland- Altman LOA plots to display agreement between parent and teacher ratings on the SDQ conduct problems rating scale.

**Fig 2:**
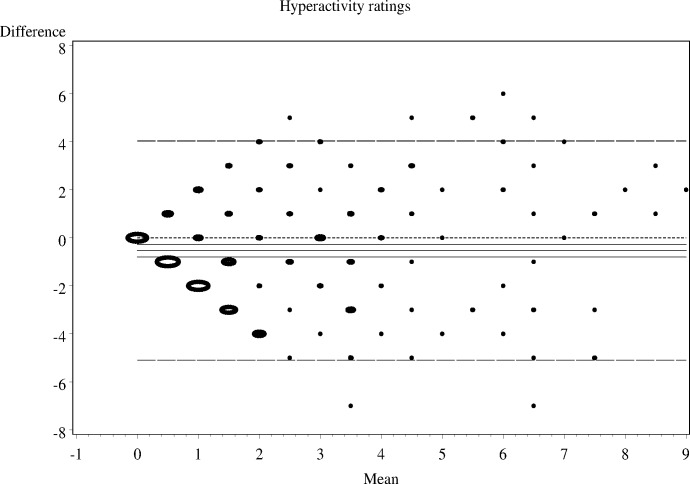
Bland- Altman LOA plots to display agreement between parent and teacher ratings on the SDQ hyperactivity rating scale.

**Fig 3:**
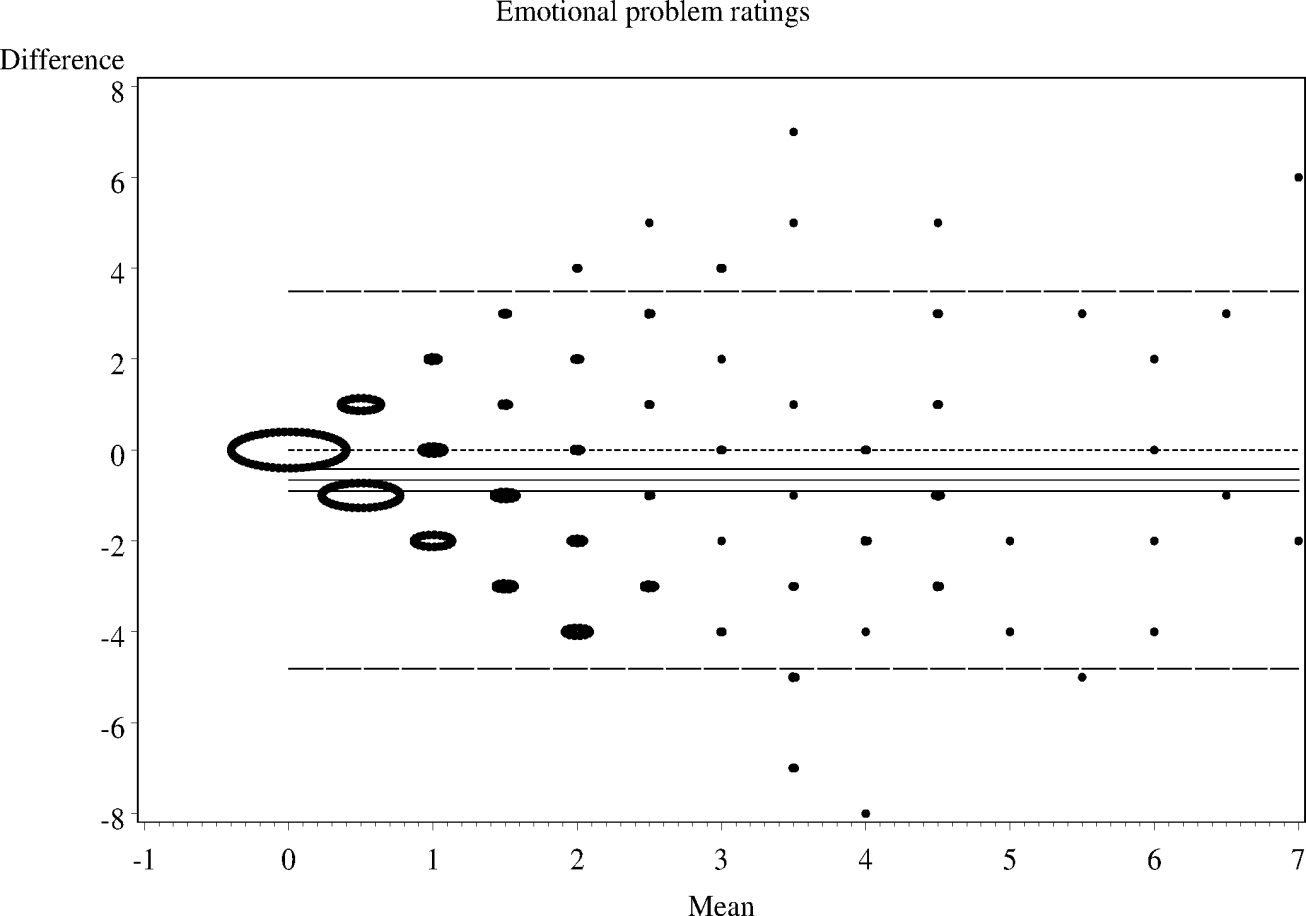
Bland- Altman LOA plots to display agreement between parent and teacher ratings on the SDQ emotional problems rating scale.

**Fig 4:**
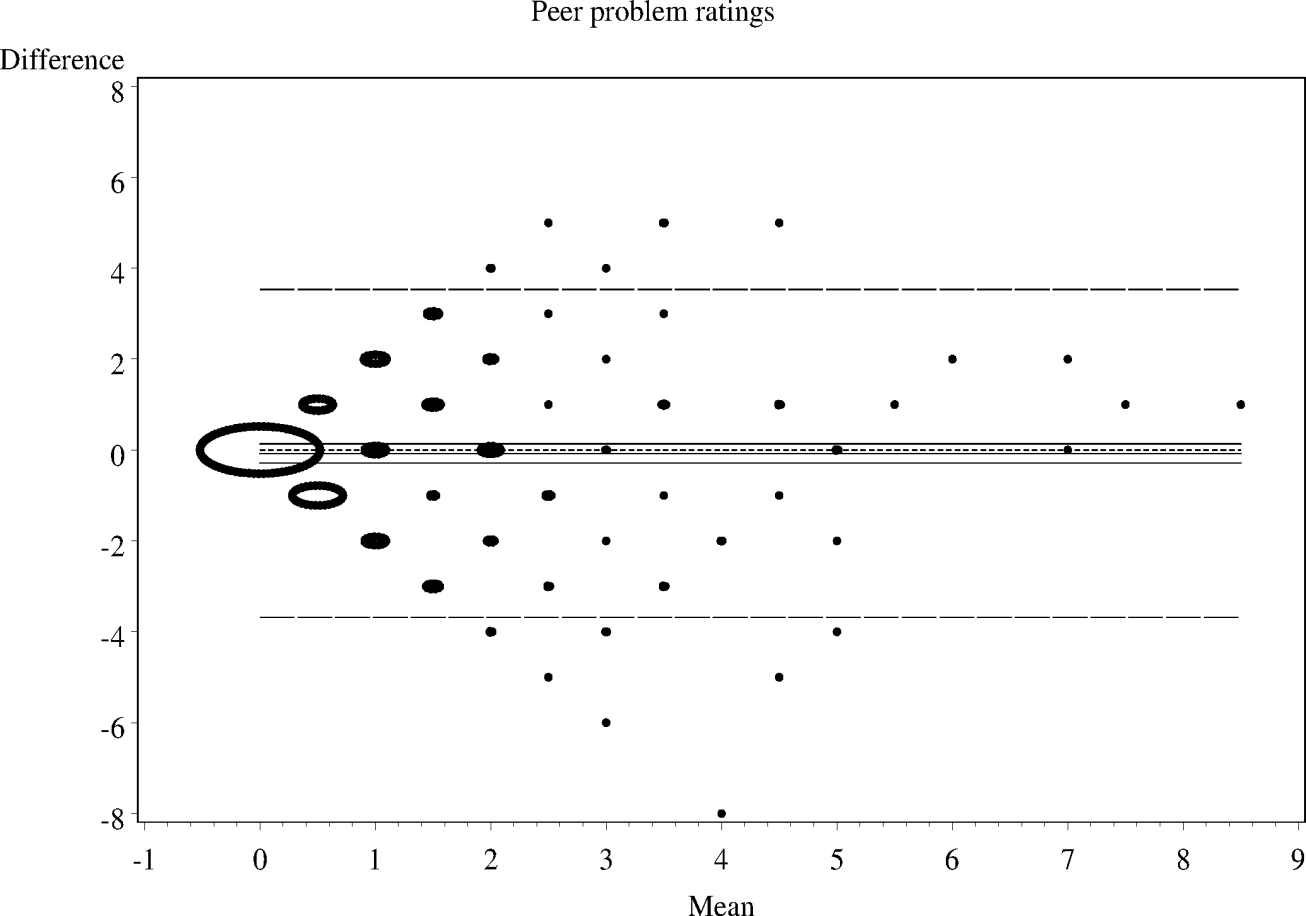
Bland- Altman LOA plots to display agreement between parent and teacher ratings on the SDQ peer problem rating scale.

**Fig 5:**
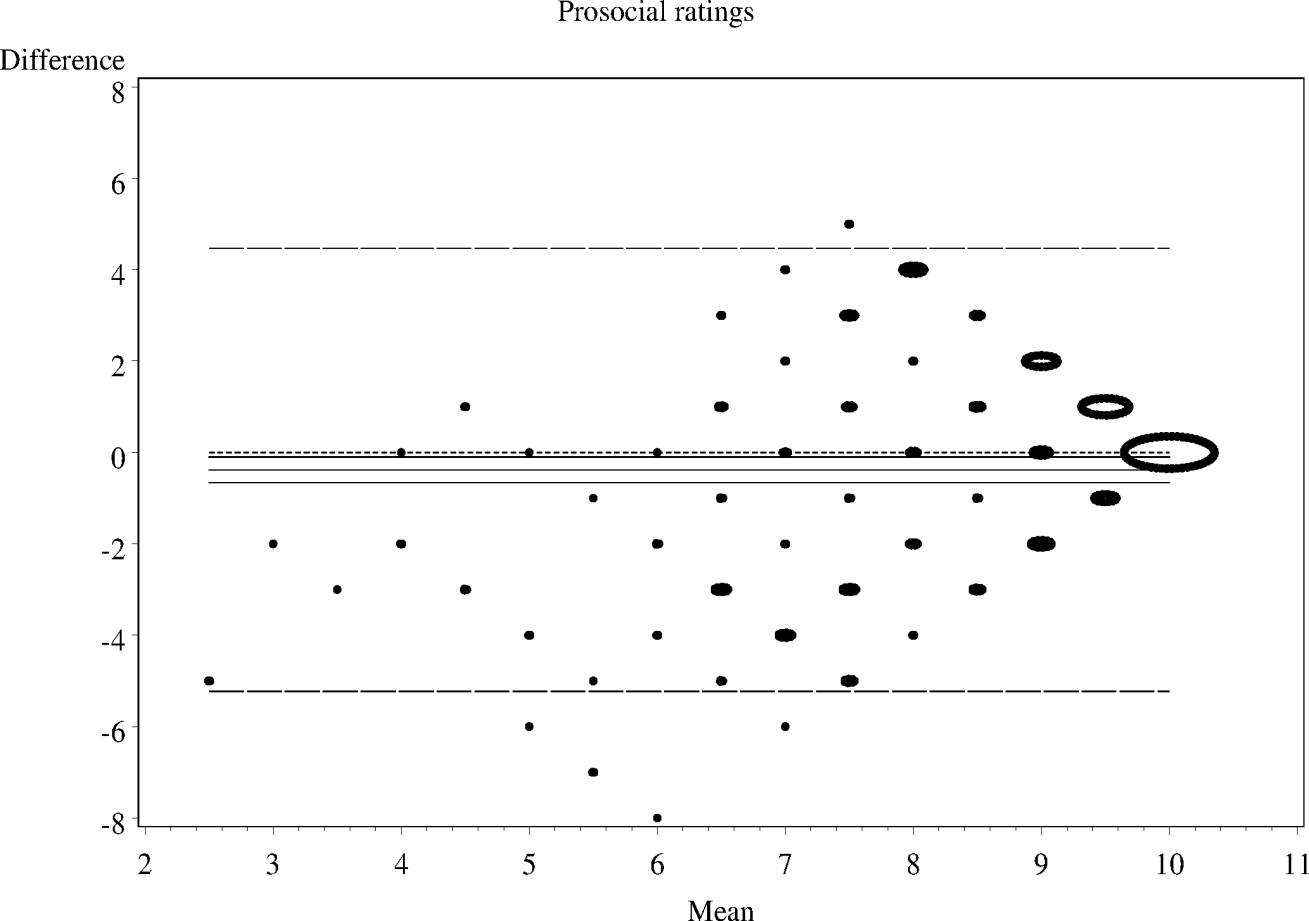
Bland- Altman LOA plots to display agreement between parent and teacher ratings on the SDQ prosocial rating scale.

**Fig 6:**
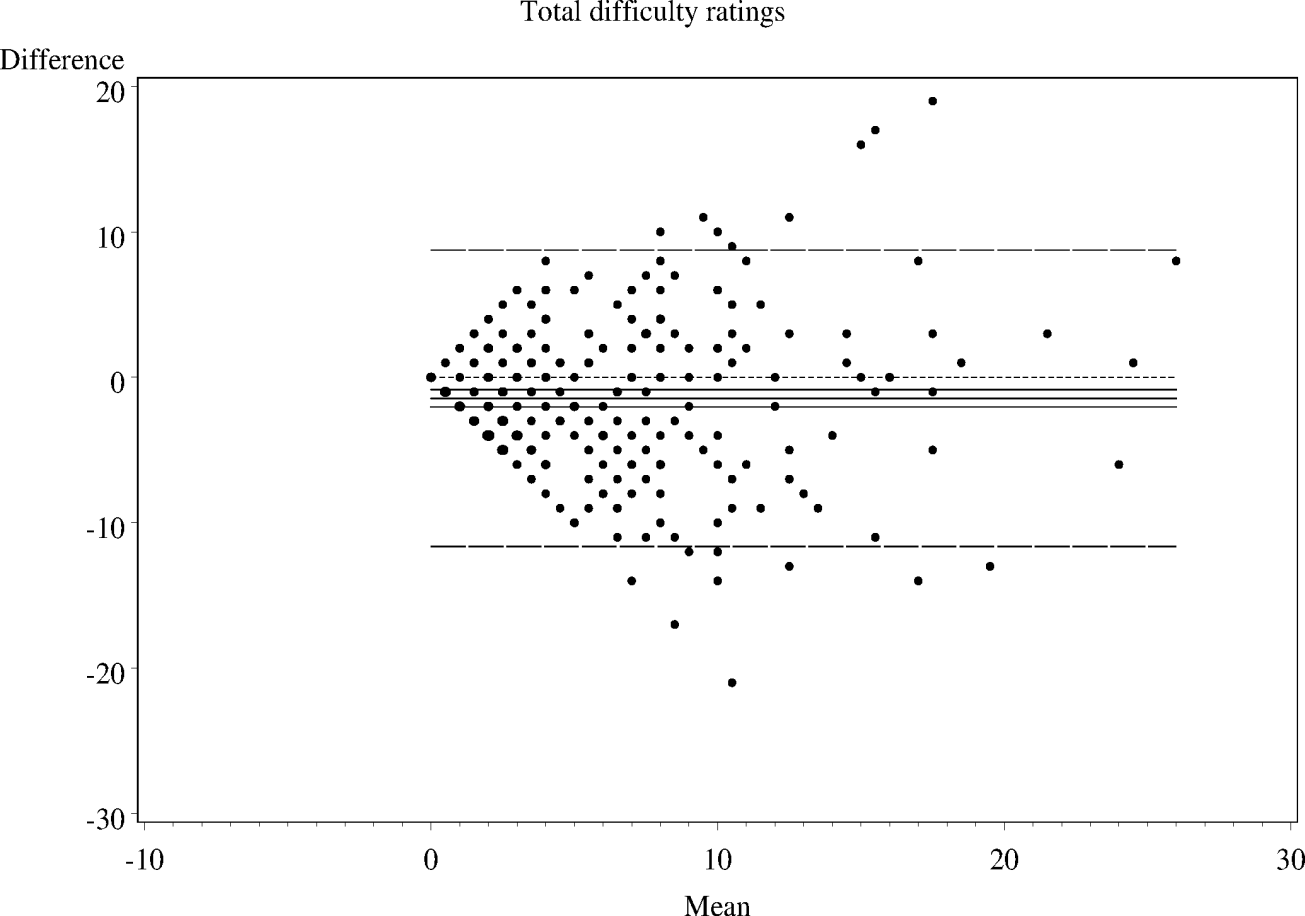
Bland- Altman LOA plots to display agreement between parent and teacher ratings on the SDQ prosocial rating scale.

As shown in Figs 1–6, the Bland-Altman plots are organised so that the frequencies of data points on each particular dot on the graph are represented by the diameter of the ring around each point (larger number of points corresponds to a ring with a larger diameter). The horizontal lines on the graph show the mean bias (the middle solid line) with its 95% confidence interval. The upper and lower LOA are represented by the dashed lines, while the dotted line shows the line of complete agreement. In many cases, the confidence interval for the bias excludes the dotted line, suggesting a consistent bias. The width of the LOA indicates the degree of discrepancy between the teacher and parent ratings.

The Bland—Altman plots showed:

- systematic differences in teacher and parent ratings on the hyperactivity and emotional scale ratings; and
- a linear relationship between measurement errors as estimated by differences in the size of the measurement for conduct problems, peer problems, and total difficulty ratings.

Given the existence of systematic error and wide LOA relative to the range of scores, the agreement between informants was explored using the possible (80% dichotomisation) and probable (90% dichotomisation) systems (Table 2).

**Table 2:** The nature of agreement between teacher and parent ratings using the Bland—Altman LOA plots.

SDQ domain	Δ Mean (SE)	95% CI of Δ Mean	95% Lower LOA (95% CI)	95% Upper LOA (95% CI)
**Conduct problems**	-0.2 (0.08)	-0.33 to -0.22	-2.9 (-3.14 to -2.60)	2.5 (2.25 to 2.79)
**Hyperactivity-inattention**	-0.5 (0.13)	-0.80 to -0.27	-5.1 (-5.55 to -4.64)	4.0 (3.57 to 4.48)
**Emotional problems**	-0.7 (0.12)	-0.90 to -042	-4.8 (-5.23 to -4.40)	3.5 (3.08 to 3.91)
**Peer problems**	-0.1 (0.11)	-0.29 to 0.13	-3.7 (-4.04 to -3.33)	3.5 (3.17 to 3.89)
**Total difficulties**	-1.4 (0.30)	-2.04 to -0.86	-11.6 (-12.66 to -10.63)	8.7 (7.73 to 9.76)

Δ—Difference; LOA = Limits of Agreement; CI = Confidence Interval

### Intraclass correlation coefficient (ICC, Absolute agreement)

The ICC between parent and teacher ratings of children’s mental health using the SDQ at the person level was .44 (95% CI: .34–.53). This showed fair parent-teacher inter-rater reliability at the individual child level. The comparable ICC calculated at item level was .96 (95% CI: .91–.98); suggesting excellent parent-teacher inter-rater reliability for SDQ items.

### Percentage of agreement, and overall classification accuracy index at item level, using the raw data

By using the parents’ ratings of the child’s mental health as the reference category, the percentage of teachers who obtained the same ratings as the parents were computed, using raw data (Table 3).

**Table 3:** Item- level agreement between parent and teacher ratings on the SDQ using raw data.

No	Type of item	Domain*	N	Proportion of teachers’ scores that agreed with parents’ scores			AC (%)	Weighted Kappa(95% CI)
				0	1	2		
**1**	Considerate of other people’s feelings	PS	299	70.64 (n = 154)	50.62 (n = 41)	0.00 (n = 0)	65.21	0.25 (0.15, 0.35)
**2**	Restless, overactive, cannot stay still for long	HI	299	82.80 (n = 183)	25.42 (n = 15)	10.53 (n = 2)	66.89	0.22 (0.12, 0.32)
**3**	Often complains of headaches, stomach aches or sickness	E	297	95.95 (n = 213)	19.35 (n = 12)	15.38 (n = 2)	76.43	0.23 (0.12, 0.35)
**4**	Shares readily with other children, e.g., toys, food	PS	298	70.47 (n = 136)	31.31 (n = 31)	0.00 (n = 0)	56.04	0.06 (-0.03, 0.17)
**5**	Easily distracted, concentration wanders	C	297	91.40 (n = 170)	11.70 (n = 11)	11.76 (n = 2)	61.62	0.11 (0.01, 0.19)
**6**	Rather solitary, prefers to play alone	PP	297	79.24 (n = 187)	21.74 (n = 10)	20.00 (n = 3)	67.34	0.12 (0.01, 0.22)
**7**	Generally well behaved, usually does what adults request	C	298	83.33 (n = 190)	36.76 (n = 25)	0.00 (n = 0)	72.15	0.31 (0.20, 0.41)
**8**	Has worries or often seems worried	E	295	84.32 (n = 156)	18.48 (n = 17)	11.11 (n = 2)	59.32	0.16 (0.06, 0.26)
**9**	Helpful if someone is hurt, upset or feeling ill	PS	299	69.12 (n = 150)	31.58 (n = 24)	33.33 (n = 1)	59.12	0.09 (-0.01, 0.20)
**10**	Constantly fidgeting or squirming	HI	297	90.87 (n = 219)	20.00 (n = 9)	9.09 (n = 1)	77.10	0.21 (0.10, 0.33)
**11**	Has at least one good friend	PP	297	88.84 (n = 223)	15.38 (n = 6)	14.29 (n = 1)	77.44	0.20 (0.07, 0.32)
**12**	Often fights with other children or bullies them	C	297	85.52 (n = 254)	35.29 (n = 6)	0.00 (n = 0)	87.54	0.28 (0.14, 0.43)
**13**	Often unhappy, depressed or tearful	E	298	91.90 (n = 227)	30.95 (n = 13)	22.22 (n = 2)	81.21	0.28 (0.14, 0.41)
**14**	Generally liked by other children	PP	299	79.83 (n = 186)	33.33 (n = 20)	33.33 (n = 2)	69.57	0.28 (0.15, 0.41)
**15**	Easily distracted, concentration wanders	HI	293	83.53 (n = 137)	30.48 (n = 32)	20.83 (n = 5)	59.39	0.30 (0.21, 0.39)
**16**	Nervous or clingy in new situations, easily loses confidence	E	297	70.34 (n = 102)	35.65 (n = 41)	13.51 (n = 5)	49.83	0.17 (0.08, 0.26)
**17**	Kind to younger children	PS	297	78.19 (n = 190)	16.67 (n = 9)	0.00 (n = 0)	67.00	0.01 (-0.10, 0.11)
**18**	Often lies or cheats	C	299	93.63 (n = 250)	22.58 (n = 7)	0.00 (n = 0)	85.95	0.18 (0.04, 0.33)
**19**	Picked on or bullied by other children	PP	298	91.70 (n = 199)	20.97 (n = 13)	15.79 (n = 3)	72.15	0.21 (0.07, 0.32)
**20**	Often volunteers to help others (parents, teachers, other children)	PS	299	41.67 (n = 104)	37.28 (n = 44)	61.54 (n = 5)	51.17	0.14 (0.04, 0.24)
**21**	Thinks things out before acting	HI	296	55.81 (n = 48)	43.96 (n = 80)	25.00 (n = 7)	45.61	0.11 (0.02, 0.20)
**22**	Steals from home or school or elsewhere	C	297	99.31 (n = 290)	50.00 (n = 2)	0.00 (n = 0)	98.32	0.39 (-0.01, 0.79)
**23**	Gets along better with adults than other children	PP	293	83.33 (n = 175)	34.32 (n = 23)	18.75 (n = 3)	68.60	0.26 (0.15, 0.37)
**24**	Has many fears, easily scared	E	297	89.27 (n = 208)	3.43 (n = 10)	10.00 (n = 1)	73.73	0.11 (-0.01, 0.22)
**25**	Good attention span, sees chores or homework through to the end	HI	296	77.05 (n = 94)	39.73 (n = 58)	28.57 (n = 8)	54.05	0.28 (0.20, 0.37)

Notes: AC = Agreed classification; *C = Conduct problems; E = Emotional problems; HI = Hyperactivity-inattention; PP = Peer problems; PS = Pro-social behaviour*

*all pro-social items are reverse coded

As shown in Table 3, item level agreement of teachers’ scores relative to parents ranged from 98.32% (item 22) to 45.61% (item 21). Eleven of the 25 items (items 3, 7, 10, 11, 12, 14, 18, 19, 22, 24) had agreement scores greater than 70%. Inter-rater agreement values for items 16 and 21were both less than 50%.

Overall, parents and teachers agreed on scoring the child as *‘not having a particular behaviour in question (rating of 0)’*–with over 90% agreement in scores found for seven of 25 items (items 3, 5, 10, 13, 18, 19, 22), and less than 50% agreement found on items 20 and 21. Agreement between parents’ and teachers’ ratings of symptom severity was less than 50% for almost all items (except items 1 and 20). Weighted Kappa coefficients were in the poor to fair category, with three items having Kappa values over 0.3 (items 7, 15, 22).

### Cohen’s weighted Kappa coefficient, percentage of agreement, and overall classification accuracy index at scale level (using the “possible or 80% dichotomisation system”)

Given that we administered the SDQ to a community sample, where the majority of students were assumed to have no mental health problems, we were interested in determining whether the 80% (Table 4) or 90% (Table 5) dichotomisation system would be most beneficial to identify cases at risk for further assessment. In all situations, the parents’ rating of the child’s mental health was treated as the true report; and the percentage of teachers who obtained the same rating as parents were computed (Tables 4 and 5). Cohen’s weighted Kappa, percentage of agreement and index of overall agreed classification accuracy indices were computed to this end.

**Table 4:** Descriptive overview of SDQ domain caseness based on parent and teacher ratings, and agreement / screening efficiency of teacher ratings relative to parent ratings using “*the possible 80% dichotomisation system”* (N = 299).

SDQ categories	Parent rating		Teacher rating		Agreement of teacher rating RT parent rating	Screening efficiency of teacher rating relative to parent rating at scale level using 80% dichotomisation								
	n	%	n	%	Kappa	Proportions			Predictive values			Likelihood/odds		
					(95% CI)	SN	SP	AC	PR	PPV	NPV	LR^+^	LR^-^	OR^D^ (95%CI)
**Conduct problems**	42	10.6	27	9.0	0.36 (0.19, 0.53)	0.40	0.94	0.88	0.10	0.46	0.93	7.17	0.63	11.28 (3.59, 35.89)
**Hyperactivity-inattention**	38	9.6	26	8.7	0.31 (0.13, 0.48)	0.35	0.94	0.88	0.09	0.39	0.93	6.04	0.68	8.85 (2.73, 29.01)
**Emotional problems**	82	20.8	18	6.0	0.18 (0.05, 0.30)	0.16	0.96	0.80	0.20	0.56	0.81	4.87	0.86	5.63 (1.65, 19.22)
**Peer problems**	90	22.8	29	9.7	0.31 (0.17, 0.44)	0.29	0.95	0.81	0.22	0.65	0.82	6.38	0.73	8.63 (3.02, 24.58)
**Total difficulties**	52	13.2	36	12.0	0.35 (0.19, 0.51)	0.44	0.92	0.86	0.13	0.45	0.91	5.56	0.60	9.17 (3.23, 26.20)

*Notes*: AC = Agreed Classification (used to represent agreed classification of teacher ratings relative to parent); LR^+^ = Positive likelihood ratio; LR^-^ = Negative likelihood ratio; PR = Prevalence; RT = relative to; SN = Sensitivity; SP = Specificity; OR^D^ = Odds ratio

**Table 5:** Scale level agreement between teacher and parent ratings on the SDQ using probable (90%) dichotomisation system.

SDQ categories	Parent rating		Teacher rating		Agreement of teacher rating RT parent rating	Screening efficiency of teacher rating relative to parent rating at scale level using 90% dichotomisation								
	n	%	n	%	Kappa(95% CI)	Proportions			Predictive values			Likelihood/odds		
						SN	SP	AC	PR	PPV	NPV	LR^+^ (95% CI)	LR^-^	OR^D^ (95% CI)
**Conduct problems**	12	4.0	16	5.4	0.32 (0.09, 0.56)	0.42	0.96	0.94	0.04	0.33	0.97	10.87 (3.20, 30.03)	0.60	17.92 (3.66, 0.97)
**Hyperactivity-inattention**	22	7.4	19	6.4	0.29 (0.09, 0.48)	0.31	0.95	0.88	0.07	0.36	0.94	7.35 (2.44, 19.68)	0.71	10.30 (2.71, 40.09)
**Emotional problems**	32	10.7	12	4.0	0.17 (0.01, 0.34)	0.15	0.97	0.88	0.11	0.42	0.90	5.96 (1.52, 21.80)	0.86	6.87 (1.56, 30.75)
**Peerproblems**	34	11.4	19	6.4	0.40 (0.23, 0.57)	0.35	0.97	0.90	0.12	0.65	0.91	13.36 (4.44, 38.92)	0.66	20.10 (5.47, 73.59)
**Total difficulties**	15	5.0	16	5.4	0.35 (0.12, 0.57)	0.37	0.96	0.93	0.07	0.45	0.95	11.79 (3.54, 34.36)	0.64	18.26 (4.07, 84.75)

Notes: AC = Agreed Classification (Used to represent agreed classification of teacher ratings relative to parent); LR^+^ = Positive likelihood ratio; LR^-^ = Negative likelihood ratio; PR = Prevalence; RT = relative to; SN = Sensitivity; SP = Specificity; OR^D^ = Odds ratio

An overview of the number and percentage of caseness identified by teacher and parent ratings on the SDQ subscales and total SDQ score is presented in Table 4, using the ‘possible’ dichotomisation system. The overall agreement between teacher and parent ratings was in the poor to moderate category (Kappa = .18–.36). Across the board, parents were more likely to identify their child as having problems that impacted on their overall functioning.

The conduct disorders category had a LR^+^ value indicative of a potentially useful test. Therefore, based on parent and teacher reports, the likelihood of a positive test result in a child having a conduct disorder was 7.17 times larger than in a chid without a conduct disorder, which is high enough to be interpreted as having the potential to alter clinical decisions. None of the categories were in the LR^-^ interval for potentially useful tests. That means that the likelihood of a child without a diagnosis having a negative test result was too low to be interpreted as having a potential to alter clinical decisions. None of the SDQ categories had OR^D^ in the range for potentially useful tests, as indicated by recommended guidelines [65]. Given the limited usefulness of teacher and parent ratings on the other SDQ domains and total SDQ scores, using the *“possible or 80% dichotomisation system”;* further analyses were conducted to determine whether a more stringent categorisation (90% dichotomisation) could improve the screening efficiency of the tool.

### Cohen’s weighted Kappa coefficient, percentage of agreement, and overall classification accuracy index at the scale level (using the “probable or 90% dichotomisation system”)

Table 5 presents a descriptive overview of SDQ domain caseness based on parent and teacher ratings, and agreement/ screening efficiency of teacher ratings relative to parent ratings using the probable (90%) dichotomisation system (N = 299)

As shown in Table 5, the overall agreement between teacher and parent ratings was poor (Kappa = .17–.40). Teachers noted a higher proportion of students to have conduct problems. Parents on the other hand found a higher percentage of students to have peer, emotional, and hyperactive problems that impacted on the overall functioning of the child. When using the 'probable' dichotomisation, the categories: peer problems; conduct disorders; hyperactive-inattention; and total difficulties were all in the LR^+^ range for potentially useful tests. This means that when there is agreement between parent and teacher reports (as reflected by LR^+^ values, the likelihood of a child being at risk of those problem behaviours after a positive test was between 7.35 and 13.36 times more likely than in a child without identified problem behaviours. None of the categories were in the LR^-^ range for potentially useful tests. The OR^D^ result for the peer problem category was in the range for potentially useful tests as indicated by recommended guidelines [65]. Given the clinical usefulness of the *“probable or 90% dichotomisation system”*, further analyses were undertaken to explicitly identify individual SDQ items that had the largest potential to alter clinical decisions.

### Screening efficiency of teachers’ ratings relative to parents’ ratings using the “probable or 90% dichotomisation” at the item level

Using the ‘probable’ dichotomisation system at item level, agreement between teachers’ and parents’ ratings improved (compare results presented in Table 6 against results presented in Table 3).

**Table 6:** Item level agreement between teacher and parent ratings on the SDQ using probable (90%) dichotomisation.

No	Item	D	N	% of agreement between teacher and parents rating		Proportions			Predictive values		Likelihood/odds		
				0 and 1	2	SN	SP	AC	PPV	NPV	LR^+(95% CI)^	LR^-^	OR^D(95% CI)^
**1**	Considerate of other people’s feelings	PS	299	70.5 (n = 154)	60.5 (n = 49)	0.61	0.71	0.68	0.42	0.83	2.06	9.55	3.68
**2**	Restless, overactive, cannot stay still for long	HI	299	96.1 (n = 269)	10.5 (n = 2)	0.10	0.96	0.90	0.14	0.94	2.67	0.93	2.87 (0.46, 18.95
**3**	Often complains of headaches, stomach aches or sickness	E	297	97.9 (n = 278)	15.5 (n = 2)	0.15	0.97	0.94	0.24	0.96	7.28*^(1.18, 37.23)^	0.86	8.424 (1.19 64.35)
**4**	Shares readily with other children, for example toys, food	PS	298	70.5 (n = 136)	37.1 (n = 39)	0.37	0.70	0.58	0.41	0.66	1.25	0.89	1.41 (0.73, 2.72)
**5**	Easily distracted, concentration wanders	E	298	98.2 (n = 275)	11.8 (n = 2)	0.11	0.98	0.93	0.30	0.94	6.58*^(1.03, 36.99)^	0.89	7.33 (1.03, 55.02)
**6**	Rather solitary, prefers to play alone	PP	297	92.2 (n = 260)	20.0 (n = 3)	0.20	0.92	0.88	1.32	0.95	2.56	0.86	2.95 (0.59, 15.16)
**7**	Generally well behaved, usually does what adults request	C	298	95.6 (n = 283)	0.0 (n = 0)	0.00	0.95	0.95	0.00	0.99	0.00	1.04	0.00 (0.00, 79.92)
**8**	Has many worries or often seems worried	E	295	95.6 (n = 265)	11.1 (n = 2)	0.11	0.95	0.90	0.13	0.94	2.56	0.92	2.76 (0.44, 18.11)
**9**	Helpful if someone is hurt, upset or feeling ill	PS	296	69.1 (n = 150)	41.8 (n = 33)	0.41	0.69	0.61	0.32	0.76	1.35	0.84	1.60 (0.80, 3.21)
**10**	Constantly fidgeting or squirming	HI	297	96.9 (n = 277)	9.1 (n = 1)	0.09	0.96	0.93	0.11	0.95	2.89	0.93	3.07 (0.30, 34.23)
**11**	Has at least one good friend	PP	297	97.6 (n = 28)	14.3 (n = 1)	0.14	0.97	0.95	0.13	0.97	5.91	0.87	6.73 (0.62, 84.00)
**12**	Often fights with other children or bullies them	C	297	98.3 (n = 291)	0.0 (n = 0)	0.00	0.98	0.98	0.00	0.99	0.00	1.01	0.00 (0.00, 57.26)
**13**	Often unhappy, depressed or tearful	E	298	97.2 (n = 281)	22.2 (n = 2)	0.22	0.97	0.95	0.18	0.97	8.02*^(1.38, 34.92)^	0.80	10.03 (1.40, 9.16)
**14**	Generally liked by other children	PP	299	96.2 (n = 282)	33.3 (n = 2)	0.33	0.96	0.95	0.20	0.98	8.87*^(1.62, 31.09)^	0.69	12.81 (1.66, 5.02)
**15**	Easily distracted, concentration wanders	HI	293	92.9 (n = 250)	20.8 (n = 5)	0.20	0.92	0.94	0.23	0.92	2.95	0.85	3.46 (0.90, 13.64)
**16**	Nervous or clingy in new situations, easily loses confidence	E	297	94.6 (n = 246)	13.5 (n = 5)	0.13	0.94	0.84	0.26	0.88	2.51	0.91	2.74 (0.71, 10.68)
**17**	Kind to younger children	PS	297	78.2 (n = 190)	22.2 (n = 12)	0.22	0.78	0.68	0.16	0.84	1.01	0.99	1.02 (0.41, 2.56)
**18**	Often lies or cheats	C	299	98.7 (n = 294)	0. 0 (n = 0)	0.00	0.98	0.98	0.00	0.99	0.00	1.01	0.00 (0.00, 603.88)
**19**	Picked on or bullied by other children	PP	298	97.1 (n = 271)	15.8 (n = 3)	0.15	0.97	0.91	0.31	0.93	5.50	0.86	6.35 (1.17, 35.91)
**20**	Often volunteers to help others (parents, teachers, other children)	PS	299	61.5 (n = 104)	52.3 (n = 68)	0.52	0.61	0.57	0.49	0.64	1.36	0.77	1.75 (0.95, 3.22)
**21**	Thinks things out before acting	HI	296	88.4 (n = 237)	25.0 (n = 7)	0.25	0.88	0.82	0.21	0.90	2.16	0.84	2.54 (0.78, 8.35)
**22**	Steals from home or school or elsewhere	C	297	100 (n = 296)	0.0 (n = 0)	-	-	0.99	-	-	-	-	-
**23**	Gets along better with adults than other children	PP	293	95.7 (n = 265)	18.8 (n = 3)	0.18	0.95	0.91	0.18	0.95	4.32	0.84	5.09 (0.98, 27.85)
**24**	Has many fears, easily scared	E	297	98.3 (n = 282)	10.0 (n = 1)	0.10	0.98	0.95	0.16	0.97	5.74	0.91	6.26 (0.58, 76.42)
**25**	Good attention span, sees chores or homework through to the end	HI	296	94.0 (n = 25)	28.6 (n = 8)	0.28	0.94	0.87	0.35	0.91	4.78	0.76	6.30 (1.87, 21.57)

*Notes*: AC = Agreed Classification (Used to represent agreed classification of teacher ratings relative to parent); LR^+^ = Positive likelihood ratio; LR^-^ = Negative likelihood ratio; PR = Prevalence; RT = relative to; SN = Sensitivity; SP = Specificity; OR^D^ = Odds ratio

Overall, 19 of the 25 items (items 1, 2, 3, 4, 7, 9, 10, 11, 12, 13, 14, 17, 18, 19, 20, 22, 23, 24) showed agreement scores greater than 70%. Agreement between parents and teachers about the level of intensity of symptoms continued to be less than chance (i.e., < 50%).

Four items: item 3 = often complains of headaches, stomach aches or sickness (emotional domain); item 5 = easily distracted, concentration wanders (emotional domain); item 13 = often unhappy, depressed or tearful (emotional domain); and item 14 = generally liked by other children (peer problems domain) were in the LR^+^ range for potentially useful tests (items in this instance). This means that the likelihood of a child having a positive test index that warrants further investigation when parents and teachers flag these items as being of concern is between 7.28–8.87 times higher than chance would occur in an individual without the condition.

## Discussion

The SDQ is one of the most common screening tools used in both educational clinical settings to flag potential mental health problems in children and adolescents [7,28]. The tool’s originator suggests that the SDQ can be used for screening; as part of a clinical assessment; as a treatment outcome measure; and as a research tool [27,46]. A recent study questioned the reliability of some of the subscales of the SDQ [28]. The current study aimed to examine the inter-rater agreement and screening concordance of the parent and teacher versions of the SDQ at scale, subscale and item levels to determine if some items have the potential to influence clinical decision making.

### Internal consistency estimates using parent and teacher forms

The raw SDQ scores of the sample were better that the Australian population norms for the 7–17 year old age group [51,53]. Consistent with the review by Stone et al. [28], the internal consistencies for several subscales failed to meet the recommended threshold for reliable use in a community sample. Whilst the ICCs between parent and teacher ratings of children’s mental health using the SDQ at the person level was fair for individual children; the comparable ICC calculated at item level was excellent, suggesting that the SDQ is reliable.

Screening is often the first step in determining who is eligible for further assessment, and can be used to identify those who are likely to benefit from immediate interventions because they are considered to be at risk [66,67]. The utility of a mental health screener may vary due to the prevalence of a disorder; and the population or setting (clinical versus community) [8,38]. A clinical population is likely to have a higher prevalence of psychosocial problems than a community population. Therefore, when used in a clinical population, the SDQ should inform us about types of problems, their duration, and perception of impact. A community population is likely to have only a few people with psychosocial problems; hence, the SDQ should be very sensitive in detecting those in the community who are at-risk of having psychosocial problems [28].

### Congruence between parent and teacher reports on the SDQ

Consistent with previous research [28], the results suggest that using the SDQ with parents only or teachers only is not recommended. Excellent parent teacher inter-rater reliability values were recorded at the item level; however, this was only the case for children demonstrating no problems on an item (i.e., a score of 0). Congruence between parents and teachers for children demonstrating any behaviour (i.e., a score of 1 or 2) was low. In addition, the weighted Kappa values were moderate to low. Weighted Kappa values are very sensitive to skewed distributions, as is the case in the present data, so the generally low Kappa values were expected. Even so, the overall congruence between parent and teacher reports was poor. Importantly, this was the case using both the ‘possible’ and ‘probable’ dichotomisation criteria.

An important consideration is the positive predictive value (PPV) value, which reflects the proportion of cases where both the parent and teacher were in agreement that the child has probable or possible mental health problems. The PPV is determined by the sensitivity and specificity of the test and prevalence; in this case the number of children identified by parents to have problems on any item at either of the two dichotomisation levels. Because the prevalence was generally low (ranging from 5–10% in most cases), a PPV of 0.4 would be considered acceptable [68]. Using the *“possible or 80% dichotomisation”*, PPV values were acceptable for the total difficulties score, as well as the conduct problems, emotional symptoms, and peer problems subscales. At the *“probable or 90% dichotomisation”* level, the PPV values were acceptable for the total difficulties score and the emotional symptoms and peer problems subscales. Given that the study comprised a community sample, within which the prevalence rate of mental health was at most 14%, the use of the *80% dichotomisation* was most appropriate. However, when using the *90% dichotomisation* at item level, most PPVs were significantly lower; suggesting that where a child is reported to have a problem on an item level by the parent (as a reference point) he or she is unlikely to be reported as having the same problem by the teacher. It should be noted, however, that the NPVs were generally very high, suggesting that the parent was not reporting a problem at the item level, which the teacher is very likely to agree. Therefore, consistent with the basic patterns of parent-teacher congruence, parents and teachers had excellent agreement when the child did not have emotional and behavioural problems. Unfortunately, the level of agreement deteriorated dramatically when parents and teachers rated their children as being *somewhat* and *certainly sure* of exhibiting emotional and behavioural difficulties. So the issue at hand relates to the false positives (i.e., the parents reported a potential mental health problem but the teacher did not). The pertinent question that follows is: “*Is a low PPV problematic in screening tools*?” Given that the SDQ was designed to identify children at risk of mental health problems, the low PPV and sensitivity means that the measure is not optimised for use in this community sample for identifying at risk children and youth. Further research examining the optimal contexts within which parents’ versus teachers’ report problem behaviours as an indicator of potential mental health problems is warranted.

### Clinical Utility of the SDQ

At both the domain and item level the specificity of the SDQ was excellent; however, sensitivity was generally poor. Given that the SDQ has been designed as a screening tool to identify at risk children [27,36], this poor sensitivity is concerning. The sensitivity of teacher and parent concordance in the current study are however similar to those reported in prior studies involving community samples [36]. Although well established, sensitivity and specificity have some deficiencies in clinical use. This is mainly due to the fact that sensitivity and specificity are population measures; that is, they summarise the characteristics of the test across a population [69]. The present study computed ratios to obtain: a) the likelihood of the adolescent having a mental health problem given a positive test result by both parents and teachers (positive likelihood ratio, LR^+^); and b) the negative likelihood ratio or the likelihood of the adolescent to not have a mental health problem given a negative test result by both parents and teachers (negative likelihood ratio, LR^-^). A LR^+^ value of 7 or greater is generally indicative of the clinical utility of a scale or item [65]. Using the *80% dichotomisation*, only the conduct problems subscale, and none of the individual SDQ items, reached this threshold. However, the total difficulties score and the subscales (with the exception of emotional symptoms) reached the threshold for clinical utility at the *90% dichotomisation* level. Moreover, three individual items also had LR^+^ scores indicating clinical utility: item 3 (often complains of headaches, stomach aches or sickness), item 13 (often unhappy, depressed or tearful), and item 14 (generally liked by other children; negative coded item). Specifically, if these items are flagged by both the teacher and parent this may indicate the probable presence of mental health problems that warrant further assessment.

Taken together, these findings suggest that when evaluating concordance between parent and teacher reports on the SDQ, using the *90% dichotomisation* system has the most clinical utility, at scale, subscale and item levels. However, this was only the case when there was agreement between parent and teacher reports (as reflected by LR^+^ values). Additionally, LR^+^ scores did not reach the threshold of 7 for the emotional symptoms subscale. This may reflect that the internalising symptoms may be difficult for both parents and teachers to observe [39]. Based on the current findings, if only parent or teacher report versions of the SDQ were administered in community samples, it appears unlikely that the SDQ will reliably identify children at risk of mental health problems. Future research should examine the clinical utility of the self-report version of the SDQ with regard to emotional symptoms, as well as examining the clinical utility of various combinations of reports to best identify students at risk of mental health problems.

## Limitations and Directions for Future Research

The current study had some major limitations which should be noted. First, the data was cross-sectional. The SDQ was the only mental health measure administered and clinical assessments were not conducted. Therefore, it was not possible to determine whether the teacher or parent report was a better indicator of child mental health and behaviour problems than clinical observations, or whether this differed as a function of symptomatology (e.g., internalising vs. externalising). Future research should examine this using prospective study designs that incorporate both a clinical assessment and administer additional outcome measures. Secondly, the sample was over-represented by students from higher mean school socio-economic sectors, which may limit the generalisation of the findings to the wider population of Australian school children. Thirdly, missing values were replaced using mean values as recommended by the SDQ developers. Using the mean value replacement technique could have resulted in biased estimates and specifically underestimated the standard errors [70]. Expectation maximisation has been recommended to overcome some of the limitation of mean substitution and should be used in the future. Also, the self-report version of the SDQ was not completed by the children in the study. Teachers and parents may not identify internalising symptoms unless they manifest in some form of observable behaviour [39]. Consistent with this, the emotional symptoms subscale was the only subscale that did not meet the LR^+^ threshold. Future research should examine concordance between parent, teacher, and youth reports on the SDQ in community samples and compare the utility of these in identifying potential mental health problems.

## Conclusion

The SDQ is one of the most widely used screening tools internationally in both clinical and community samples. Consistent with a recent review [28], internal consistencies did not reach recommended thresholds for the total difficulties score (teacher report), as well as the conduct problems (parent report), peer problems (both parent and teacher reports), and prosocial behaviour (parent report) subscales. Moreover, given that the purpose of the SDQ as a screening measure is to identify children at risk of mental health and behavioural problems, the low PPVs is of concern.

In the current community sample, the SDQ only demonstrated clinical utility when there was agreement between teacher and parent reports using the *90% dichotomisation* system. Moreover, three individual items also had LR^+^ scores indicating clinical utility: item 3 (often complains of headaches, stomach aches or sickness), item 13 (often unhappy, depressed or tearful), and item 14 (generally liked by other children). Specifically, if these items are flagged by both teacher and parent this may indicate the probable presence of mental health problems and warrant further assessment. Further research is needed to learn more about the relationship of items to each other and the contribution of each item to its subscale score and its contribution to the overall difficulties score. Of note was the finding that the negative likelihood ratio or likelihood of disregarding the absence of a condition when both parents and teachers rated the item as absent was not significant. There is a need for further research to identify in which contexts parent and teacher reports might independently show clinical utility. Taken together, these findings suggest that the SDQ is not optimised for use in community samples and that further psychometric evaluation of the SDQ in this context is clearly warranted.
